# A2B adenosine receptor-triggered intracellular calcium mobilization: Cell type-dependent involvement of Gi, Gq, Gs proteins and protein kinase C

**DOI:** 10.21203/rs.3.rs-5442142/v1

**Published:** 2024-12-13

**Authors:** Zhan-Guo Gao, Ray R. Gao, Clayton K. Meyer, Kenneth A. Jacobson

**Affiliations:** NIDDK, National Institutes of Health; NIDDK, National Institutes of Health; NIDDK, National Institutes of Health; NIDDK, National Institutes of Health

**Keywords:** A2B adenosine receptor, GPCR, G protein, calcium, Gq, Gi, Gs

## Abstract

Activation of PLCβ enzymes by G_iβγ_ and G_αq/11_ proteins is a common mechanism to trigger cytosolic Ca^2+^ increase. We and others reported that G_αq/11_ inhibitor FR900358 (FR) can inhibit both and G_αq_- and, surprisingly, G_iβγ_-mediated intracellular Ca^2+^ mobilization. Thus, the G_αi_-G_βγ_-PLCβ-Ca^2+^ signaling axis depends entirely on the presence of active G_αq_, which reasonably explained FR-inhibited G_iβγ_-induced Ca^2+^ release. However, the conclusion that G_iβγ_ signaling is controlled by G_αq_ derives mostly from HEK293 cells. Here we show that indeed in HEK293 cells both G_αq/11_ siRNA and G_αq/11_ inhibitors diminished Ca^2+^ increase triggered by native G_q_-coupled P2Y_1_ receptors, or by transfected G_i_-coupled A_1_-or G_s_-coupled A_2B_ adenosine receptors (ARs). However, in T24 bladder cancer cells, G_i_ inhibitor PTX, but not G_αq/11_ inhibitors, FR, YM254890 (YM) or G_q/11_ siRNA, inhibited Ca^2+^ increase triggered by native A_2B_AR activation. Simultaneous inactivation of G_i_ and G_s_ further suppressed A_2B_AR-triggered Ca^2+^ increase in T24 cells. The G_αq/11_ inhibitor YM fully and partially inhibited endogenous P2Y_1_- and β_2_-adrenergic receptor-induced Ca^2+^ increase in T24 cells, respectively. PKC activator PMA partially diminished A_2B_AR-triggered but completely diminished β_2_-adrenergic receptor-triggered Ca^2+^ increase in T24 cells. Neither β-arrestin1 nor β-arrestin2 siRNA affected A_2B_AR-mediated Ca^2+^ increase. Unlike in T24 cells, YM inhibited native A_2B_AR-triggered calcium mobilization in MDA-MB-231 breast cancer cells. Thus, G_αq/11_ is vital for Ca^2+^ increase in some cell types, but G_iβγ_-mediated Ca^2+^ signaling can be Gα_q/11_-dependent or independent based on cell type and receptor activated. Besides G proteins, PKC also modulates cytosolic Ca^2+^ increase depending on cell type and receptor.

## Introduction

Intracellular calcium mobilization triggered by G protein-coupled receptors (GPCRs), which ~ 34% of prescription drugs target [1], affects all aspects of cell functions including neurotransmitter release, insulin secretion, T cell activation, muscle contraction, immunity and cell survival and proliferation [2]. Activation of PLCβ enzymes by a GPCR is considered as a common mechanism to trigger Ca^2+^ increase in cells. Both G_iβγ_ and G_αq_, activated by G_i_-coupled and G_q_-coupled receptors, respectively, can trigger PLC activation [3] or intracellular calcium mobilization [4–7].

We previously compared the G protein-inhibitory effects of FR900358 (FR, UBO, UBO-QIC [8]) and pertussis toxin (PTX) on various GPCR signaling pathways [9]. For example, FR (UBO, at 300 nM), had no effect on A_2B_ adenosine receptor (AR)-G_αs_-mediated cAMP accumulation or A_1_AR- G_αi_-mediated inhibition of forskolin-stimulated cAMP accumulation in CHO cells [9]. Nevertheless, FR (100 nM) produced a nearly complete inhibition of G_iβγ_-mediated calcium mobilization, or IP1 accumulation, upon the activation of the G_i_-coupled A_1_AR, the M_2_ muscarinic acetylcholine receptor, or the P2Y_12_ receptor (P2Y_12_R, responds to ADP) overexpressed in either CHO or 1321N1 astrocytoma cells. However, 100 nM FR had no effect on A_1_AR-mediated Akt1/2/3 phosphorylation, which was completely inhibited by PTX.

FR (100 nM) produced a small (< 30%) but significant inhibition of A_1_AR-mediated ERK1/2 activity [9]. It is also of note that FR, at both 100 and 300 nM, only partially inhibited ERK1/2 activity triggered by the G_q/11_-coupled M_3_ acetylcholine or P2Y_1_ (responds to ADP) receptor, although the calcium response triggered by these two receptors was completely inhibited.

In a follow-up study, Pfeil et al. [10] further explored the potential mechanism related to FR-inhibited G_iβγ_-triggered calcium signaling in one signaling pathway mentioned above, i.e. calcium signaling, but not Akt or ERK1/2 signaling. They confirmed our finding [9] and suggested that “the G_αi-Gβγ_-PLCβ-Ca^2+^ signaling axis is entirely dependent on the presence of active G_αq_. If G_αq_ is pharmacologically inhibited or genetically ablated, G_αq_ can bind to PLCβ but does not elicit Ca^2+^ signals.” The finding related to G_αq/11_ control of G_iβγ_-mediated Ca^2+^ signaling is an important discovery, since the mechanisms related to Ca^2+^ increase in cells is vital to the discovery of drugs targeting different receptor classes. However, the conclusion that G_αq_ controls G_iβγ_-mediated Ca^2+^ increases via removal of PLC auto-inhibition was drawn mostly from the study of native and G protein-knockout HEK293 cells [10].

We have previously shown the critical role of G_αq/11_ in A_2B_AR-triggered calcium mobilization in HEK293 cells overexpressing the recombinant human (h) A_2B_AR [12,13]. In A_2B_AR-overexpressing HEK293 cells, both CRISPR-Cas9-based G_q/11_ knockout and the G_q_ inhibitor FR completely eliminated the A_2B_AR-mediated Ca^2+^ increase [12,13]. It is of note that the native A_2B_AR in HEK293 cells is capable of inducing cAMP accumulation via G_s_ and ERK1/2 activity increase via G_i_, but it is unable to trigger a robust Ca^2+^ signal [12,13]. However, when hA_2B_AR is overexpressed in HEK293 cells, a robust intracellular Ca^2+^ mobilization signal mediated via G_αq/11_ is induced [12–14], which suggests that A_2B_AR overexpression could enable the coupling to G_αq/11_.

The A_2B_AR has emerged as an important target for various conditions, including cancer, cardiac ischemia and sarcopenia [15–20]. Calcium signaling is a major signaling pathway involved in these critical conditions. Thus, it is important to better understand the A_2B_AR downstream signaling, including Ca^2+^ mobilization. The cytosolic Ca^2+^ increase triggered by the nonselective AR agonist NECA has been explored using T24 bladder cancer cells expressing the native hA_2B_AR [12,21]. NECA-induced Ca^2+^ increase in T24 cells can be completely blocked by a selective A_2B_AR antagonist PSB603 and diminished by A_2B_AR siRNA [12]. In addition, Panjehpour et al. [22] reported that a NECA-triggered Ca^2+^ signal in MDA-MB-231 breast cancer cells also occurs via the A_2B_AR. In the present study we further examined the mechanisms of A_2B_AR-triggered Ca^2+^ release, especially whether G_iβγ_-triggered Ca^2+^ release can be blocked by G_αq/11_ siRNA or pharmacological inhibitors, in HEK293 cells overexpressing the recombinant A_2B_AR, and in T24 bladder cancer cells expressing a native A_2B_AR. Thus, we examined the effects of two G_αq/11_ inhibitors: FR and YM-254890 (YM [23]), as well as G_αq/11_ siRNA, the G_i_ inactivator PTX, and the G_s_ inactivator CTX. We found that, unlike in HEK293 cells, G_iβγ_, but not G_q/11_, plays a critical role in A_2B_AR-triggered Ca^2+^ increase in T24 bladder cancer cells. We also analyzed the role of G_s_ (relative to G_i_) in A_2B_AR-mediated Ca^2+^ release, the synergistic effects between G_i_ and G_s_, and cell type- and receptor-dependent modulation by protein kinase C (PKC). In some cases, we compared the A_2B_AR-mediated effects with those of other purinergic receptors or biogenic amine receptor.

## Materials and Methods

### Materials

BAY60–6583 (LUF6210) was provided by Prof. Ad IJzerman at Leiden-Amsterdam Center for Drug Research (Leiden, The Netherlands). Nonselective AR agonist adenosine-5′-*N*-ethyluronamide (NECA), β_2_ adrenergic receptor agonists formoterol and isoproterenol, lysophosphatidic acid (LPA) receptor agonist LPA, YM254890, PSB603, ESI-09, H89, PKC activator phorbol 12-myristate 13-acetate (PMA), and PKC inhibitor Go6983 were from Tocris (Minneapolis, MN, USA). FR900359 (UBO-QIC) was purchased from University of Bonn (Germany). HEK293 cells, T24 bladder cancer cells, and MDA-MB-231 cells were from ATCC (Manassas, VA, USA). HEK293-A2B cells were made at the Laboratory of Bioorganic Chemistry, NIDDK, NIH (Bethesda, MD, USA). An AlphaScreen cAMP kit was purchased from PerkinElmer (Waltham, MA). Calcium assay kits were from Molecular Devices (Sunnyvale, CA, USA). All other reagents were from standard commercial sources and of analytical grade.

### RNA extraction and quantitative real-time PCR detection of gene expression level

Total RNA was extracted from 10^7^ cells using a RNeasy kit (Qiagen, Redwood City, CA, USA) and was reversed-transcribed using the SuperScript^™^ III First-Strand Synthesis SuperMix kit (ThermoFisher, Waltham, MA, USA) according to the manufacturer’s protocol and as described [24]. Briefly, the thermocycles were as follows: 25°C for 10 min; 50°C for 30 min; 85°C for 5 min and then chilled on ice; 1 μl (2 U) of E Coli RNase H was added and incubation continued for 20 min before storage at −20°C until use. The cDNA was then amplified with TaqMan gene expression assays (ThermoFisher, Waltham, MA USA) for four AR subtypes and GAPDH on a CFX96 Touch Real-Time PCR Detection System (BioRad, Hercules, CA, USA) according to the manufacturer’s protocol. The temperature cycles were: 50°C for 2min; 95°C for 10 min; 95°C 15s and 60°C for 30s for 40 cycles. The ΔΔCt method was used to conduct quantitative analysis of data. Values were normalized to GAPDH and then expressed as relative expression levels.

### Cell culture and measurement of cyclic AMP levels

HEK293, T24 bladder cancer and MDA-MB-231 breast cell lines were cultured in DMEM medium containing 10% fetal bovine serum, 100 units/ml penicillin, 100 μg/ml streptomycin, and 2 μmol/ml glutamine. For the assay of 3′,5′-cyclic adenosine monophosphate (cAMP), cells were plated in 96-well (4×10^4^ cells/well) clear plates in 100 μl of medium overnight. Cell culture medium was then replaced with 80 μl HBSS buffer containing 20 mM HEPES, phosphodiesterase inhibitor rolipram (10 μM), and 3 units/ml adenosine deaminase (Worthington Biochemical, Lakewood, NJ, USA) for 30 min followed by agonist addition to the mixture, which was then incubated for 20 min. The treatment of YM or FR was 20 min before agonist addition. The treatment with CTX (500 ng/ml or PTX (200 ng/ml) was for overnight before agonist addition. The reaction was terminated by aspirating the reaction mixture and the addition of 100 μl cold 0.3% Tween-20 to each well. Cells were then shaken at room temperature for 10 min. For the determination of cAMP production, an AlphaScreen cAMP kit was used according to the manufacturer’s instructions (Revvity/PerkinElmer, Waltham, MA, USA).

### Measurement of intracellular calcium increase

The measurement of calcium mobilization was essentially as described previously [19,24]. We utilized a calcium assay kit as directed without washing cells and with probenecid added to the loading dye at a final concentration of 2.5 mM to increase dye retention. Briefly, cells were grown at 37°C/5% CO_2_ in a set of 96-well black-wall, clear-bottom plates overnight, or until they reach confluence. Then, media was aspirated, and 100 μl of dye (Calcium 6) was added to each well. Afterward, the plates were subsequently maintained at room temperature in the dark for 60 min. Finally, 50 μl of compound or a control agonist was added into respective assay plate wells during the determination of intracellular Ca^2+^ using a FLIPR (Molecular Devices, San Jose, CA, USA). The treatment of YM and FR was 20 min before agonist addition. The treatment with CTX (500 ng/ml) or PTX (200 ng/ml) was for overnight before agonist addition. The compound plate was prepared using dilutions of various compounds in HBSS buffer (pH 7.4) without added calcium. Samples were run in duplicate or triplicate at room temperature. Cell fluorescence (excitation = 485 nm; emission = 525 nm) was monitored following compound exposure. Increases in intracellular Ca^2+^ are reported as the maximum fluorescence value after exposure minus the basal fluorescence value before exposure.

### Statistical and data analyses

Functional parameters were calculated using Prism 10.2.3 software (GraphPad, San Diego, CA, USA). Data was expressed as mean ± standard error. A Student’s *t-test* (between two conditions) or a One-Way Analysis of Variance (ANOVA) followed by Tukey’s or Bonferroni’s multiple comparison tests (between multiple conditions) was used to compare statistically significant differences. Differences yielding *P* values < 0.05 are considered statistically significant.

## Results

### HEK293 cells: G_αq/11_ protein contribution to A_2B_AR-mediated intracellular calcium mobilization

**Figure 1:**
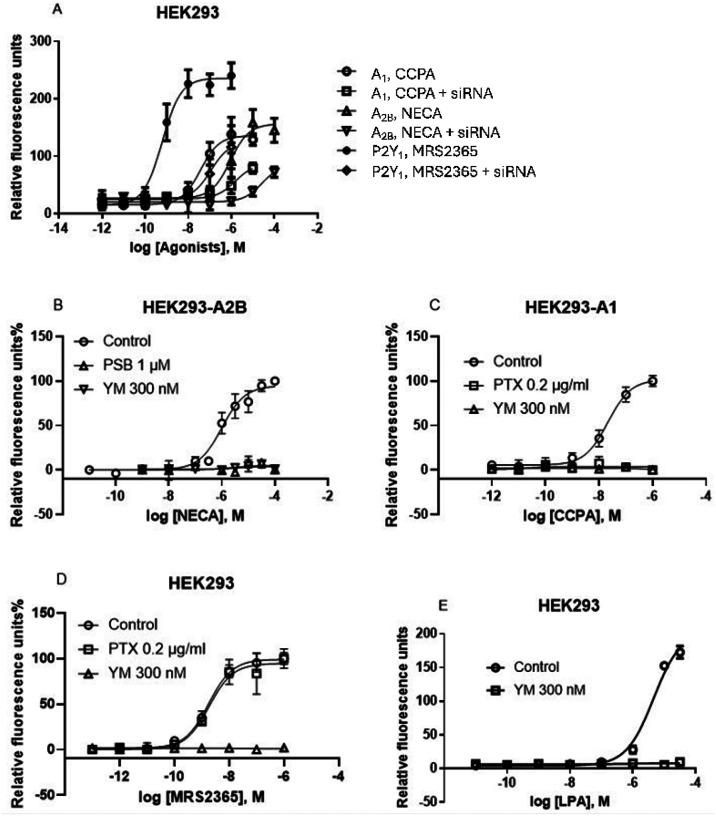
G_q/11_ siRNA knockdown and effects of G_q/11_ inhibitors in HEK293 cells. A. Effects of G_q/11_ siRNA (250 nM) on A_2B_-, A_1_-, and P2Y_1_ receptor-mediated intracellular calcium mobilization in HEK293 cells. Data are mean ± SEM from three independent experiments. B,C,D,E. The recombinant A_1_ and A_2B_ARs are stably expressed in HEK293 cells. The P2Y_1_ and LPA receptors are endogenously expressed in HEK293 cells. CCPA, A_1_ agonist; NECA, A_2B_AR agonist; MRS2365, P2Y_1_R agonist. Data expressed as mean ± SEM from 2–4 independent experiments.

Here, we further explore the G_αi_-G_βγ_-PLCβ-Ca^2+^ signaling axis, previously demonstrated to be inhibited by G_αq/11_ inhibitor FR and Gq/11 knockout [10,13]. In addition to FR, G_q/11_ siRNA and another G_q/11_ inhibitor, YM, inhibited calcium mobilization in HEK293 cells, endogenously expressing the G_q_-coupled P2Y_1_R, or overexpressing the recombinant human G_i_-coupled A_1_ or G_i_- and G_s_-coupled A_2B_ ARs (Fig. 1). These findings are consistent with previous pharmacological results [9] and align with the proposed mechanism that G_iβγ_-mediated calcium signaling is under G_q/11_ control in HEK293 cells [10]. Thus, the results from the present study using FR, YM, G_q/11_ siRNA knockdown and prior CRISPR-Cas9-based G_q/11_ knockout research [10] are all consistent.

**Figure 2:**
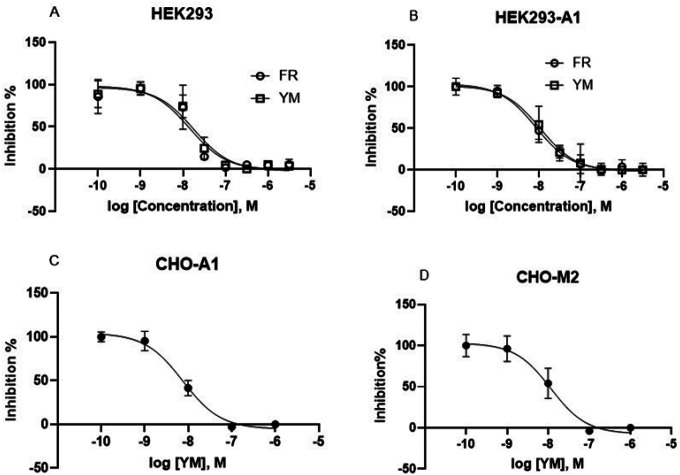
Actions of FR and YM at G_q_-coupled P2Y_1_ and G_i_ coupled A_1_ and M_2_ receptors. Potencies of FR and YM in inhibition of G_αq_- (HEK293 a native P2Y_1_R) or G_iβγ_-mediated calcium increase. A. MRS2365 (1 μM)-induced Ca^2+^ in HEK293 cells expressing a native P2Y_1_R (G_q_ coupled). B. CCPA (1 μM)-induced Ca^2+^ increase in HEK293 cells expressing the recombinant A_1_AR (G_i_-coupled). C. CCPA (1 μM)-induced Ca^2+^ increase in CHO cells expressing the recombinant A_1_AR. D. Carbachol (10 μM)-induced Ca^2+^ increase in CHO cells expressing the recombinant M_2_ muscarinic receptors. Data are from at three independent experiments. The IC_50_ values (nM) are listed in the text.

Unlike the G_q_ inhibitors FR and YM, treatment with the G_i_ inhibitor PTX had no effect on G_q/11_-coupled P2Y_1_R agonist MRS2365-induced Ca^2+^ release in HEK293 cells (Fig. 2D), but completely inhibited the A_1_AR agonist CCPA-induced Ca^2+^ signaling in HEK293-A1 cells (Fig. 2C). The A_2B_AR-mediated increase of ERK1/2 activity in HEK293-A2B cells has been reported to be G_i_-dependent by Yang et al. [25] based on PTX sensitivity, which was later confirmed with both PTX and CRISPR-Cas9-based Gq/11 knockout [13]. However, A_2B_AR-mediated Ca^2+^ mobilization in HEK293-A2B cells is G_i_-independent, as incubation with 200 ng/ml PTX for 18 hours did not affect the NECA-induced Ca^2+^ increase [14].

It is of note that the native A_1_AR expression level in HEK293 cells is extremely low, and A_1_AR agonist CCPA does not trigger Ca^2+^ signaling [12,13]. The native A_2B_AR in HEK293 cells can induce robust cAMP accumulation and ERK1/2 activity but not sufficient to trigger a robust calcium signaling [12,13,19]. In HEK293 cells, there is a possibility that the overexpression of G_i_-coupled A_1_ or G_s_-coupled A_2B_ ARs enable their stronger coupling to G_αq/11_, and thus G_αq/11_ can subsequently control the A_1_AR-mediated Giβγ-Ca^2+^ signaling. FR inhibition of native G_q/11_-coupled P2Y_1_R-induced calcium release is expected. In addition, LPA (lysophosphatidic acid)-induced intracellular calcium mobilization (EC_50_ = 4.59 μM) was also completely inhibited by 300 nM YM (Fig. 2E).

#### HEK293 cells: Similar potencies of G _αq/11_ inhibitors on G_iβγ_- versus G_αq_-mediated stimulation of intracellular calcium mobilization

The A_1_AR and P2Y_1_R are G_i_-coupled and G_q_-coupled receptors, respectively. We compared the potencies of FR and YM in inhibiting A_1_AR (overexpressed)-triggered calcium mobilization by A_1_AR agonist CCPA (1 μM) and native P2Y_1_R-mediated Ca^2+^ increase by P2Y_1_R agonist MRS2365 (1 μM) in HEK293 cells (Fig. 2A,B). The IC_50_ values FR and YM to inhibit P2Y_1_R-mediated calcium mobilization were 13.2 ± 3.2 and 12.8 ± 4.1 nM, respectively. The IC_50_ values for the inhibition of the A_1_AR-mediated effect were 8.66 ± 3.23 and 12.9 ± 2.52 nM, respectively. Additionally, the IC_50_ values of YM in inhibiting the effects of 10 μM carbachol and 1 μM CCPA in CHO cells overexpressing the recombinant G_i_-coupled M_2_ muscarinic or A_1_ receptor, respectively, were determined to be 9.53 and 11.6 nM, respectively (Fig. 2C,D). Thus, the potencies of FR and YM for inhibition of G_q_- and G_iβγ_-mediated calcium signaling are similar, and the use of either inhibitor at 100–300 nM should completely inhibit both G_iβγ_- and G_αq_-triggered Ca^2+^ signaling.

### HEK293 cells: G inhibitors lack effects on G-mediated inhibition or G-mediated stimulation of cAMP accumulation

**Figure 3:**
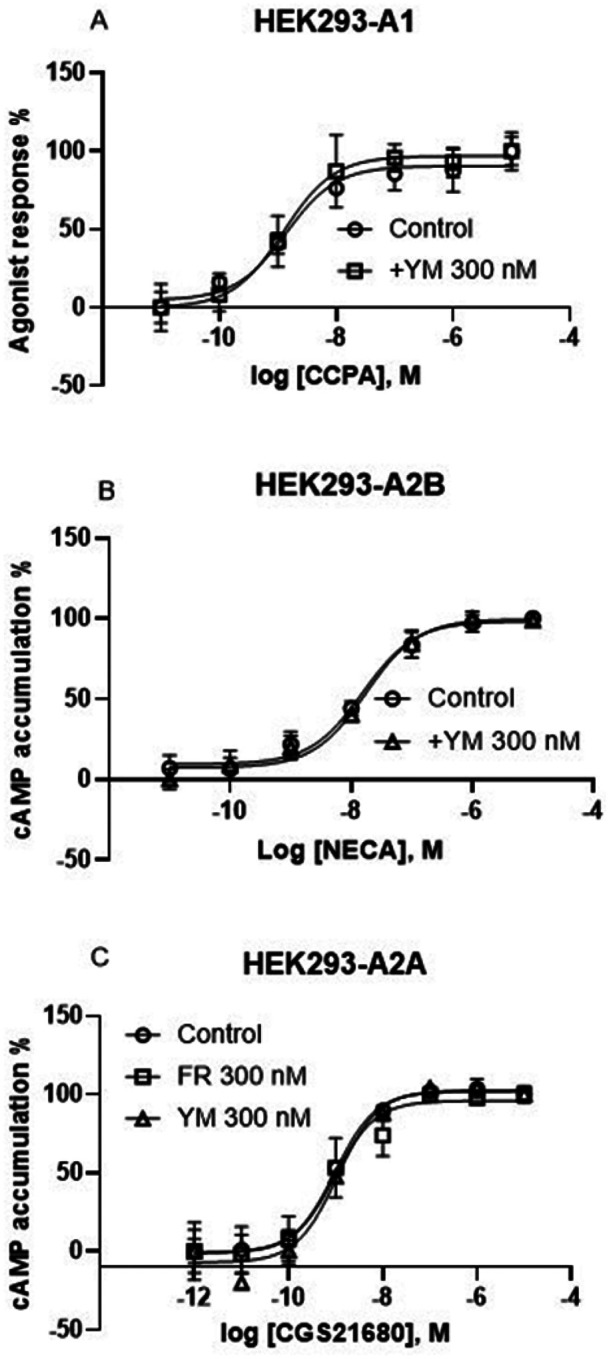
Effects of FR and/or YM on G_i_-mediated inhibition or G_s_-mediated stimulation of cAMP accumulation in HEK293 cells. Data are from three separate experiments. A. Inhibition by CCPA of forskolin-stimulated cAMP accumulation. B. NECA-induced cAMP accumulation. C. CGS21680-induced cAMP accumulation.

As previously demonstrated in other cell types [9], FR and/or YM did not affect A_1_AR-mediated inhibition or A_2A_AR- and A_2B_AR-mediated stimulation of cAMP accumulation in HEK293 cells (Fig. 3), although they inhibited Ca^2+^ signaling induced by those receptors.

#### T24 bladder cancer cells: G _q/11_ proteins do not play a major role in native A_2B_AR-mediated intracellular calcium mobilization

**Figure 4:**
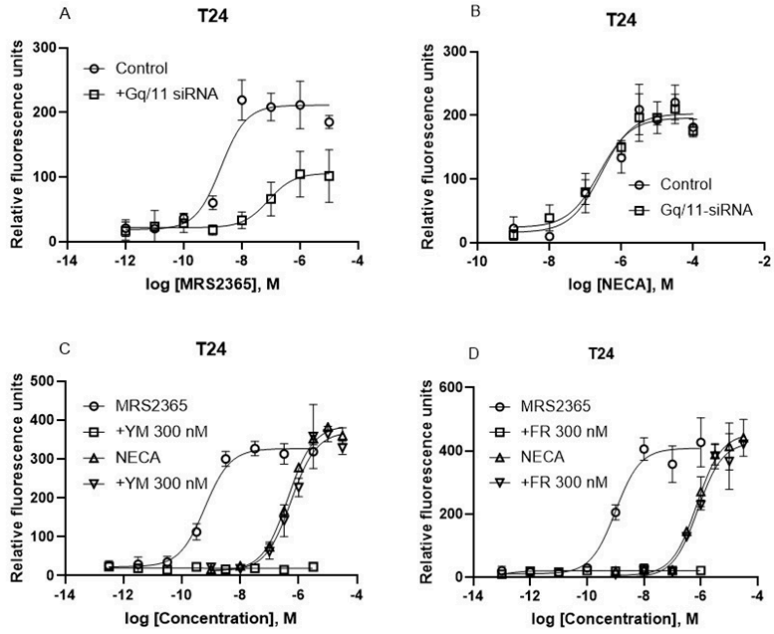
Roles of G_q/11_ proteins in intracellular calcium mobilization in endogenously expressed GPCRs in T24 cells. A,B. G_q/11_ siRNA on P2Y_1_R agonist MRS2365- and A_2B_AR agonist NECA-induced a Ca^2+^ increase mediated by native P2Y_1_ and A_2B_ receptors, respectively. C,D. The effect of G_q/11_ chemical inhibitor YM (C) FR (D). Results are from 2–4 independent experiments.

As described above, the G_q/11_ pharmacological inhibitors and siRNA diminished the A_2B_AR-mediated calcium signaling in HEK293 cells overexpressing the recombinant hA_2B_AR, which is consistent with results from CRISPR-Cas9-based G_q/11_ knockout HEK293 cells [13]. We next carefully examined the intracellular calcium mobilization triggered by the native A_2B_AR in T24 bladder cancer cells [12,21]. Figure 4A shows that, unlike in HEK293 cells, G_q/11_ siRNA suppressed P2Y_1_R agonist MRS2365-induced calcium mobilization but not A_2B_AR agonist NECA-triggered calcium mobilization (Fig. 4B). The EC_50_s of NECA in the absence and presence of G_q/11_ siRNA were 234 ± 61 and 247 ± 46 nM, respectively (n = 3), which are not significantly different (*P* > 0.05, Student’s *t-test*).

Similar to the effect of G_q/11_ siRNA, Fig. 4C shows that the G_q/11_ inhibitor YM (300 nM) also completely inhibited the effect of P2Y_1_R agonist MRS2365 but had little if any effect on A_2B_AR-induced calcium mobilization in T24 cells. The EC_50_s of NECA in the absence and presence of YM were 427 ± 76 and 524 ± 88 nM, respectively (n = 3), which are not significantly different (*P* > 0.05, Student’s *t-test*). Figure 4D shows that, similarly to YM in T24 cells, another G_q/11_ inhibitor FR (300 nM) also completely inhibited calcium mobilization triggered by the native P2Y_1_R but not by the native A_2B_AR.

#### T24 bladder cancer cells: Contribution of both G _i_ and G_s_ proteins to intracellular calcium mobilization triggered by native A_2B_ARs

**Figure 5:**
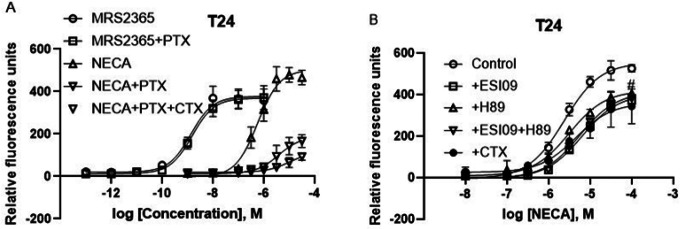
Roles of G_i_ and G_s_ proteins in intracellular calcium mobilization mediated by endogenous GPCRs in T24 cells. Data are from three independent experiments. A. Effects of G_i_ inactivator PTX (200 ng/ml) and the combination of PTX and Gs inactivator CTX (500 ng/ml). B. effects of G_s_ inactivator CTX (500 ng/ml), EPAC inhibitor ESI09 (10 μM), or PKA inhibitor H89 (10 μM).

Figure 5A shows that, unlike YM and FR, PTX diminished A_2B_AR agonist NECA-mediated calcium mobilization in T24 cells but had no effect on the P2Y_1_R agonist MRS2365. Thus, it is suggested that G_i_ plays a prominent role in A_2B_AR-mediated intracellular calcium mobilization in T24 bladder cancer cells. Considering that G_iα_ isoforms do not play a significant role in activation PLCβ [26], it is assumed that G_βγ_ is responsible for this effect (Gao and Jacobson, 2016). Interestingly, simultaneous inactivation of G_i_ and G_s_ by PTX and CTX almost completely eliminated A_2B_AR-triggered Ca^2+^ release (Fig. 5A), suggesting both G_iβγ_ and G_s_ are responsible for A_2B_AR-mediated Ca^2+^ mobilization in T24 cells. The EC_50_s of MRS2365 in the absence and presence of PTX were 1.38 ± 0.26 and 1.55 ± 0.47 nM, respectively, which are not significantly different (*P* > 0.05, Student’s *t-test*). The EC_50_s of NECA for Control group, PTX group and PTX + CTX group were 538 ± 112, 3550 ± 890 and 12,300 ± 4600 nM, respectively, which are significantly different (*P* < 0.05, One-Way Analysis of Variance followed by Bonferroni’s multiple comparison tests). The maximal effect of NECA (in terms of relative fluorescence units) in Control group, PTX group and PTX + CTX group were 505 ± 78, 173 ± 39 and 88 ± 15, respectively, which are significantly different (*P* < 0.05, One-Way Analysis of Variance followed by Bonferroni’s multiple comparison tests).

To examine whether the pathway downstream of G_s_, PKA or EPAC, contributes to A_2B_-Gs-mediated calcium mobilization in T24 cells, PKA inhibitor H89 and EPAC inhibitor ESI09 were used. Figure 5B shows that both EPAC and PKA are involved in A_2B_AR-G_s_-mediated calcium mobilization. However, the combination of ESI09 and H89 did not produce an effect larger that ESI09 alone (P > 0.05, Student’s *t-test*). The inactivation of G_s_ by CTX produced a similar effect to that of ESI09.

**Figure 6:**
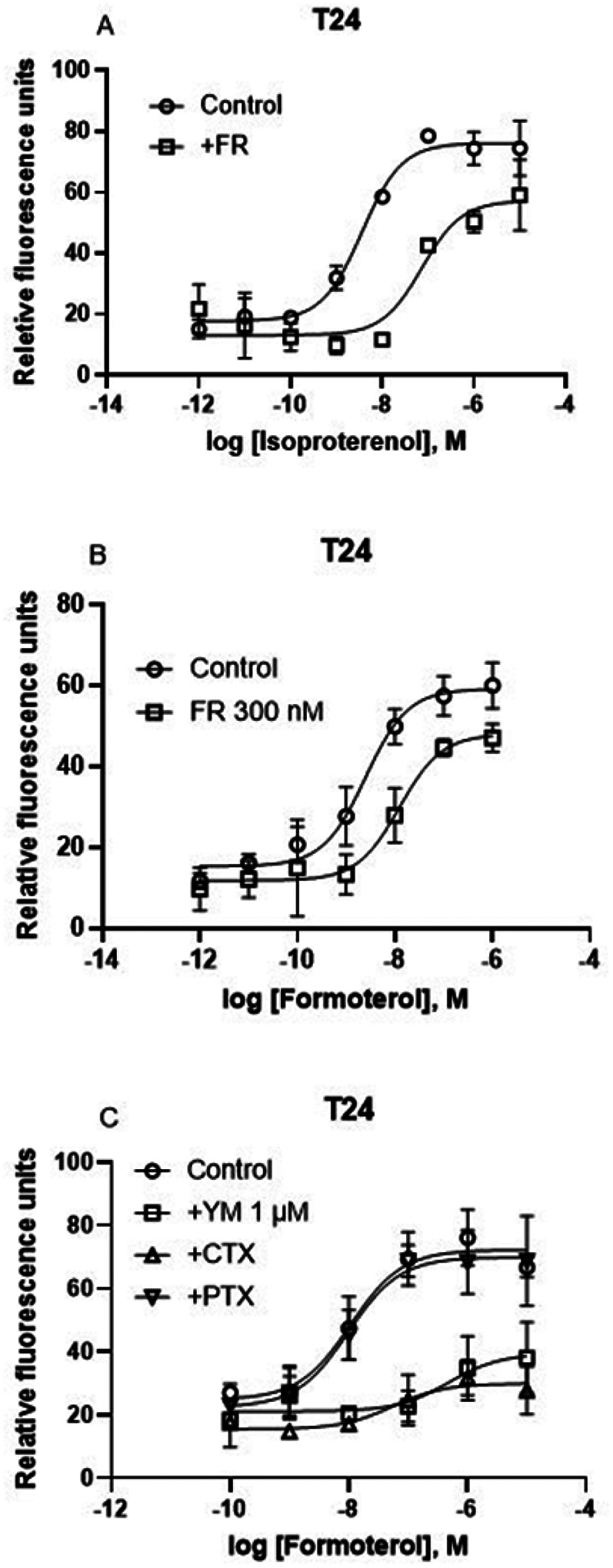
Effects of G_αq_ inhibitors FR and YM, G_i_ inactivator PTX and G_s_ inactivator CTX on endogenous GPCR-mediated Ca^2+^ increase in T24 cells. A,B. FR (300 nM) on triggered Ca^2+^ increase triggered by β-adrenergic receptor agonists isoproterenol- and formoterol in T24 cells. C. Effects of YM (1 μM), CTX (500 ng/ml) and PTX (200 ng/ml). Data are from three independent experiments.

In addition to the A_2B_AR, T24 cells also endogenously express the β_2_-adrenergic receptor. Figure 6 shows that the β_2_ adrenergic agonist formoterol and isoproterenol induced a robust calcium response albeit a little less efficacious compared to the A_2B_AR agonist NECA. However, unlike the effects of FR on the A_2B_AR agonist NECA-triggered Ca^2+^ release, FR (300 nM) diminished Ca^2+^ increase triggered by both β_2_-adrenergic receptor agonists formoterol and isoproterenol, suggesting a role of G_q/11_ (Fig. 6A,B).

In a separate set of experiments, YM (1 μM) was shown to inhibit 78% of the maximum effect (E_max_) of formoterol (Fig. 6C). Unlike its effect on the A_2B_AR, PTX treatment did not produce significant attenuation of the E_max_ in β_2_ agonist formoterol-induced calcium mobilization (Fig. 6c). However, CTX treatment produced an effect larger than it was on the NECA-induced Ca^2+^ response (Fig. 6C; Fig. 5B) in the same cell type, i.e. T24 bladder cancer cells. Thus, even in the same cell type, the involvement of G proteins in intracellular calcium mobilization is dependent on receptor type, although both the A_2B_AR and the β_2_-adrenergic receptor have been reported to couple to both G_s_ and G_i_ [13,27]. We were not able to observe formoterol-triggered Ca^2+^ increase in HEK293 cells.

**Figure 7:**
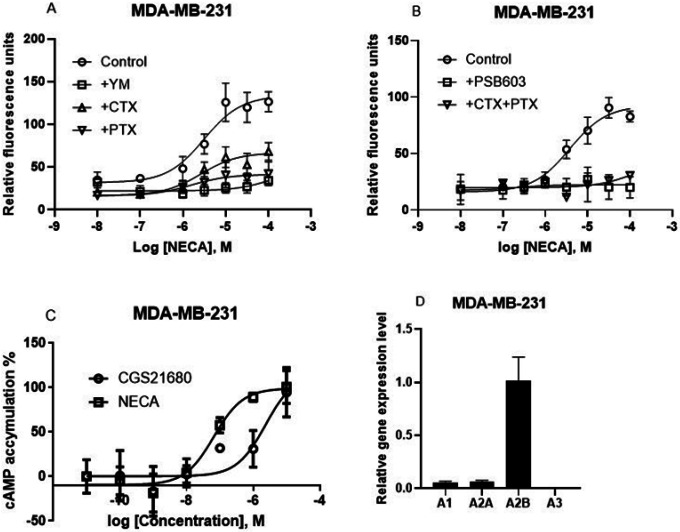
Effects on agonist-triggered Ca^2+^ increase in MDA-MB-231 breast cells expressing endogenous adenosine receptors. A. Effects of YM (300 nM), CTX (500 ng/ml) and PTX (200 ng/ml) on the response to NECA. B. Effects of A_2B_ antagonist PSB603 (1 μM) and the combination of CTX and PTX on NECA-triggered Ca^2+^ increase. C. Comparison of cAMP accumulation induced by A_2A_ agonist CGS21680 and A_2B_ agonist NECA. D. Gene expression level of four ARs on MDA-MB-231 cells. Results are from three experiments performed in duplicate.

In addition to T24 cell, the A_2B_AR has been reported to trigger intracellular calcium mobilization in a human breast cancer cell line, MDA-MB-231 [22], although the G proteins involved have not been examined. In the present study, we compared the effect of CTX, PTX and YM. Figure 7 shows that, unlike in T24 bladder cancer cells, YM, CTX, and PTX all inhibited NECA-induced Ca^2+^ increase although to a different extent. Thus, G_i_, G_s_ and G_q_ are all involved in the A_2B_AR-mediated Ca^2+^ increase in MDA-MB-231 cells. Furthermore, CTX and PTX together produced an effect larger either alone (Fig. 7B). The selective A_2B_AR antagonist PSB603 (1 μM) completely blocked the effect of NECA (Fig. 7B).

Thus, we demonstrated that intracellular calcium mobilization in two cell types, i.e. T24 bladder cancer and MDA-MB-231 breast cancer, both expressing the native A_2B_ARs, is independent and dependent on G_q/11_, respectively. A cAMP accumulation assay confirmed the A_2B_AR rather than A_2A_AR is the dominant AR subtype (Fig. 7C). The gene expression levels of four AR subtypes in MDA-MB-231 cells are shown in Fig. 7D with A_2B_AR being the highest expressed.

### T24 bladder cancer cells: Involvement of PKC in A_2B_AR-mediated intracellular calcium mobilization

**Figure 8:**
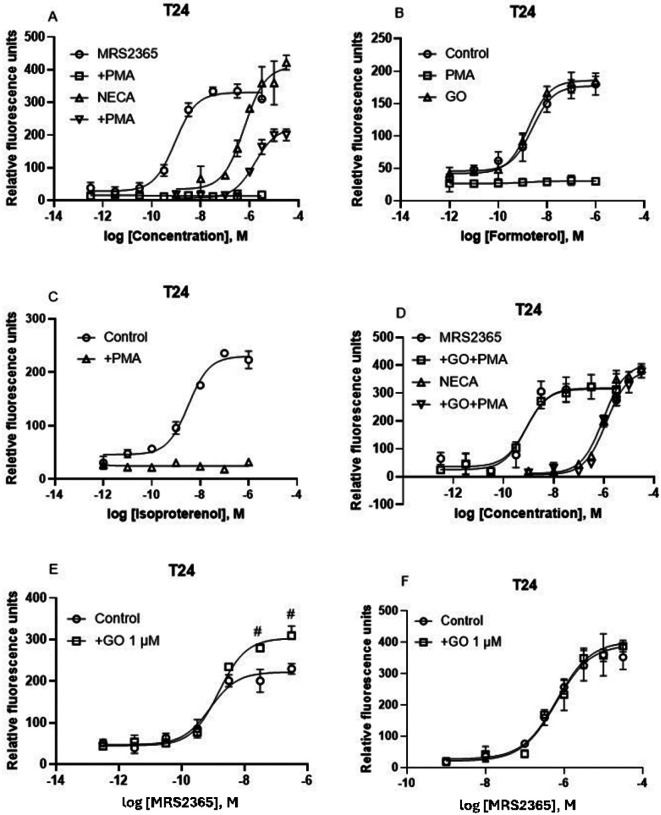
Role of protein kinase C in A_2B_AR-, P2Y_1_- and β_2_-adrenergic receptor-triggered Ca^2+^ increase in T24 cells. A. Effects of PKC activator PMA on MRS2365- or NECA-triggered Ca^2+^ increase. B. Effects of PMA or PKC blocker GO (Go6983). C. Effects of PMA. D. Effects of preincubation of GO for 20 min followed by addition of PMA for 20 min before addition of agonists. E,F. Effects of PKC inhibitor on P2Y_1_- or A_2B_- triggered Ca^2+^ increase. Results are from three experiments. #Significantly different from control (P<0.05).

We have demonstrated previously in HEK293-A2B cells that the activation of PKC by PMA completely diminished the Ca^2+^ mobilization induced by the A_2B_AR agonist NECA [19]. Figure 8 shows that in addition to G_s_, G_iβγ_, and G_αq/11_, PKC also plays a role in receptor-triggered cytosolic calcium increase. The PKC activator PMA completely eliminated P2Y_1_R-mediated calcium mobilization, but only partially diminished A_2B_AR-mediated calcium mobilization in T24 cells (Fig. 8A). Unlike the effect of PKC on the A_2B_AR, but similar to its effect on the P2Y_1_R, cell treatment with PKC activator PMA (1 μM) completely diminished the intracellular calcium mobilization induced by β_2_ agonists formoterol (Fig. 8B) and isoproterenol (Fig. 8C). Thus, the effect of PKC on calcium intracellular mobilization in T24 cells is receptor type-dependent. Pretreatment of cells with the PKC inhibitor GO for 20 min eliminated the effect of PMA at both A_2B_AR and P2Y_1_R (Fig. 8D). Interestingly, the PKC inhibitor GO alone significantly enhanced the E_max_ of calcium mobilization induced by P2Y_1_R agonist MRS2365 (*P* < 0.05, One-Way ANOVA) but not by the A_2B_AR agonist NECA, which suggests that PKC could act as an endogenous suppressor of P2Y_1_R-calcium signaling in T24 bladder cancer cells.

#### T24 bladder cancer cells: β-Arrestins 1 and 2 are not involved in A _2B_ AR-mediated intracellular calcium mobilization

**Figure 9:**
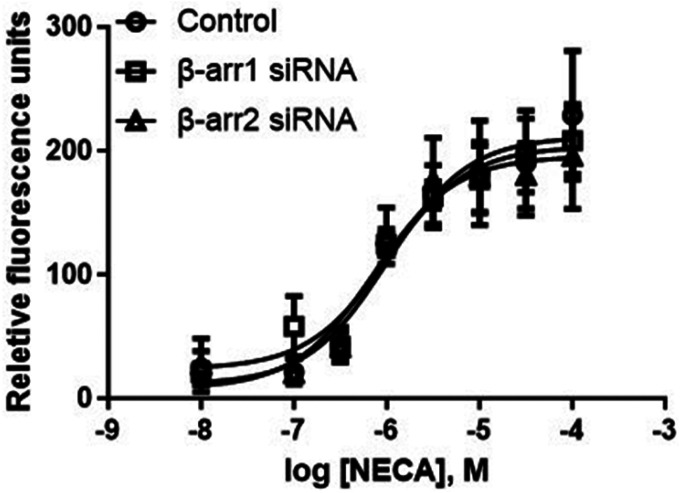
Effects of β-arrestin siRNA on A_2B_AR agonist NECA-triggered Ca^2+^ increase in T24 cells. Data are expressed as mean ± SEM from three experiments. Results are from three experiments. The transfection of siRNA was performed using Lipofectamine 2000.

We have shown previously that β-arrestin2 siRNA diminished P2Y_1_R agonist MRS2365-induced and β-arrestin2-mediated ERK1/2 activity [28]. Here it is shown that neither β-arrestin1 nor β-arrestin2 siRNA affected A2BAR agonist NECA-induced Ca^2+^ increase (Fig. 9).

### T24 bladder cancer cells: L-type calcium channels are not involved in A_2B_AR-mediated intracellular calcium mobilization

**Figure 10:**
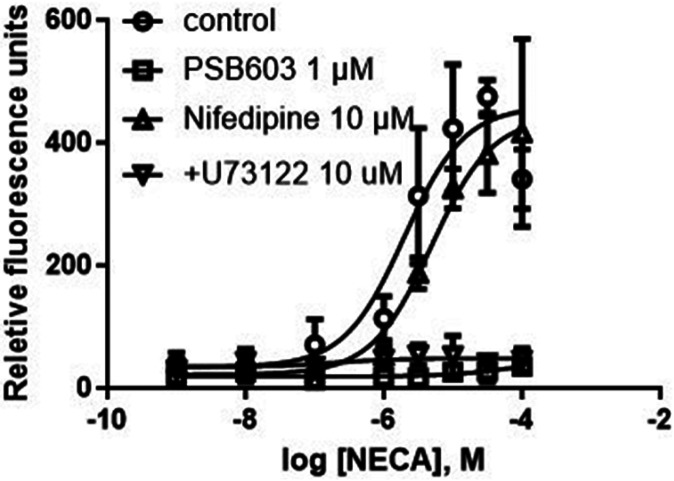
Effects of the L-type calcium channel blocker nifedipine, A_2B_ antagonist PSB603 and the PLC inhibitor U73122 on A_2B_ agonist NECA-triggered Ca^2+^ increase in T24 cells. Results are from three experiments. Inhibitors were incubated with cells for 20 min in 96-well black plates before addition of NECA.

Figure 10. Effects of the L-type calcium channel blocker nifedipine, A_2B_ antagonist PSB603 and the PLC inhibitor U73122 on A_2B_ agonist NECA-triggered Ca^2+^ increase in T24 cells. Results are from three experiments. Inhibitors were incubated with cells for 20 min in 96-well black plates before addition of NECA.

## Discussion

We have probed the mechanisms of GPCR-triggered calcium fluctuations in specific cell types, in which a GPCR is either endogenous or overexpressed, and may be coupled to G_i_-, G_s_- or G_q_-protein. In the present study, we expanded the previous work and further explored A_2B_AR-mediated calcium transients, showing that G_i_, G_s_, G_q_ and PKC play different roles in different cell types. We also compare results with the A_2B_AR, either overexpressed or endogenously expressed in HEK293 cells, with several other GPCRs (Table 1).

## Figures and Tables

**Table 1:** Purinergic (i.e. A_2B_, A_1_, and P2Y_1_) and biogenic amine receptor signaling leading to calcium mobilization, showing results of treatment with pharmacological inhibitors or activators. The related figure is shown in italic parentheses.

Modulation of G protein, arrestin or PKC - and effect					
Cell type and receptor	G_αq/11_	G_iβγ_	G_s_	β-arrestin1/2	PKC
A_2B_AR					
HEK293 (A_2B_ overexpressing)	FR and YM and Gα_q/11_ siRNA sensitive **(1)**	PTX insensitive^a^	ND	ND	PMA completely sensitive (Gao et al., 2023)
T24 (native A_2B_)	FR and YM insensitive **(4)**; Gα_q/11_ siRNA insensitive **(4)**	PTX sensitive **(5)**	CTX sensitive **(5)**, Variable, depending on cell type^b, c^	β-arrestin1 or β-arrestin2 siRNA insensitive **(9)**	PMA partially sensitive **(8)**
MDA-MB-231 (native A_2B_)	YM sensitive **(7)**	PTX partly sensitive **(7)**	CTx partly sensitive **(7)**	ND	ND
A_1_AR					
HEK293 (A_1_ overexpressing)	YM and FR – fully sensitive **(2)**	PTX sensitive **(1)**	ND	ND	ND
P2Y_1_R					
HEK293 (native P2Y_1_)	Gα_q/11_ siRNA partially sensitive **(1)**; YM and FR – fully sensitive **(2)**	PTX insensitive **(1)**	ND	ND	ND
1321N1 astrocytoma (P2Y_1_ overexpressing)^d^	YM sensitive	PTX insensitive	ND	ND	ND
T24 (native P2Y_1_)	YM – fully sensitive **(4)**	PTX insensitive **(5)**	ND	ND	ND
β_2_R^e^					
T24 (native b_2_)	YM – partially sensitive **(6)**	PTX not sensitive **(6)**	CTX sensitive **(6)**	ND	PMA completely sensitive **(8)**

a Linden et al., 1999 [14].

b NECA-induced Ca^2+^ increase in HMC-1 cells was insensitive to CTX and PTX, Feoktistov et al., 1995 [29].

c NECA-induced Ca^2+^ increase in human erythroleukemia cells was sensitive to CTX, Feoktistov et al., 1994 [30].

d Gao and Jacobson, 2017 [28].

e G_q/11_ inhibitors blocked calcium mobilization by activation of the G_i_-coupled M_2_ muscarinic receptor overexpressed in CHO cells.

ND, not determined.

## Data Availability

Primary data for this study is available from the authors upon reasonable request.

## Inclusion and Diversity

We support inclusive, diverse and equitable conduct of research.

## Abbreviations

CCPA: 2-chloro-*N*^6^-cyclopentyladenosine
CTX: cholera toxin
EPAC: exchange protein activated by cAMP
FLIPR: Fluorometric Imaging Plate Reader
FR: FR900358
GPCR: G protein-coupled receptor
GAPDH: glyceraldehyde 3-phosphate dehydrogenase
GLP: glucagon-like peptide
GRK: G protein-coupled receptor kinases
HBSS: Hanks balanced salt solution
HEPES: 2-[4-(2-hydroxyethyl)piperazin-1-yl]ethane-1-sulfonic acid
LPA: lysophosphatidic acid
NECA: adenosine-5′-*N*-ethyluronamide
PKC: protein kinase C
PLC: phospholipase C
PMA: phorbol 12-myristate 13-acetate
PTX: pertussis toxin
YM: YM254890
